# Supplementary material for: Genomics of 1 million parent lifespans implicates novel pathways and common diseases and distinguishes survival chances
Source: eLife. 2019 Jan 15;8:e39856. doi: 10.7554/eLife.39856 (PMC6333444; doi:10.7554/eLife.39856)
Supplement: Supplementary file 1. — This PDF contains a table and plots of the lifespan GWAS and eQTL signals genes from Westra and CAGE eQTL studies that pass FDR < 5% threshold for the SMR test and p>0.05 threshold for HEIDI test. [file elife-39856-supp1.pdf]

Supplementary file 1: Loci with significantly predicted candidate genes using SMR-HEIDI test and two eQTL datasets (blood tissue)

					Westra		CAGE	
At or near	rsID	Chr	Position	SMR Genes	P SMR	P HEIDI	P SMR	P HEIDI
CELSR2/PSRC1	rs4970836	1	109821797	PSRC1			7.3E-08	0.2980
FOXO3	rs3800231	6	108998266	SESN1	8.2E-04	0.2640		
ATXN2/BRAP	rs11065979	12	112059557	SH2B3	3.8E-04	0.0602		
CHRNA3/5	rs8042849	15	78817929	PSMA4	8.2E-10	0.2240	1.3E-05	0.1080
CHRNA3/5	rs8042849	15	78817929	PSMA4	3.1E-08	0.5590		
FURIN/FES	rs6224	15	91423543	FES	1.8E-06	0.0550		
FURIN/FES	rs6224	15	91423543	FURIN			1.8E-04	0.0621
LDLR	rs142158911	19	11190534	KANK2	7.2E-04	0.5660		

All 24 loci discovered or replicated in our study were tested against blood tissue eQTL data from two studies, Westra and CAGE. Only genes that pass FDR < 5% threshold for the SMR test and P > 0.05 threshold for HEIDI test are listed for their corresponding loci. At or near – nearby gene or cluster of genes to lead variant; rsID – reference SNP cluster ID for the index SNP in the region; Chr – chromosome Position – Base-pair position on chromosome (build GRCh37); SMR genes – genes prioritised by SMR within the given locus.

The following pages contain plots of the lifespan GWAS and eQTL signals. Gene expression probe names are provided with the corresponding gene names in brackets. The pSMR threshold corresponds to a significance level of FDR < 5%, and the gene expression probes that have SMR signal passing this threshold are displayed as red diamonds, otherwise blue. Filled diamonds indicate that the corresponding probes also pass the P > 0.05 threshold for the HEIDI test, i.e. the expressions of the particular genes possibly share causal variants with the lifespan GWAS signals.

Westra

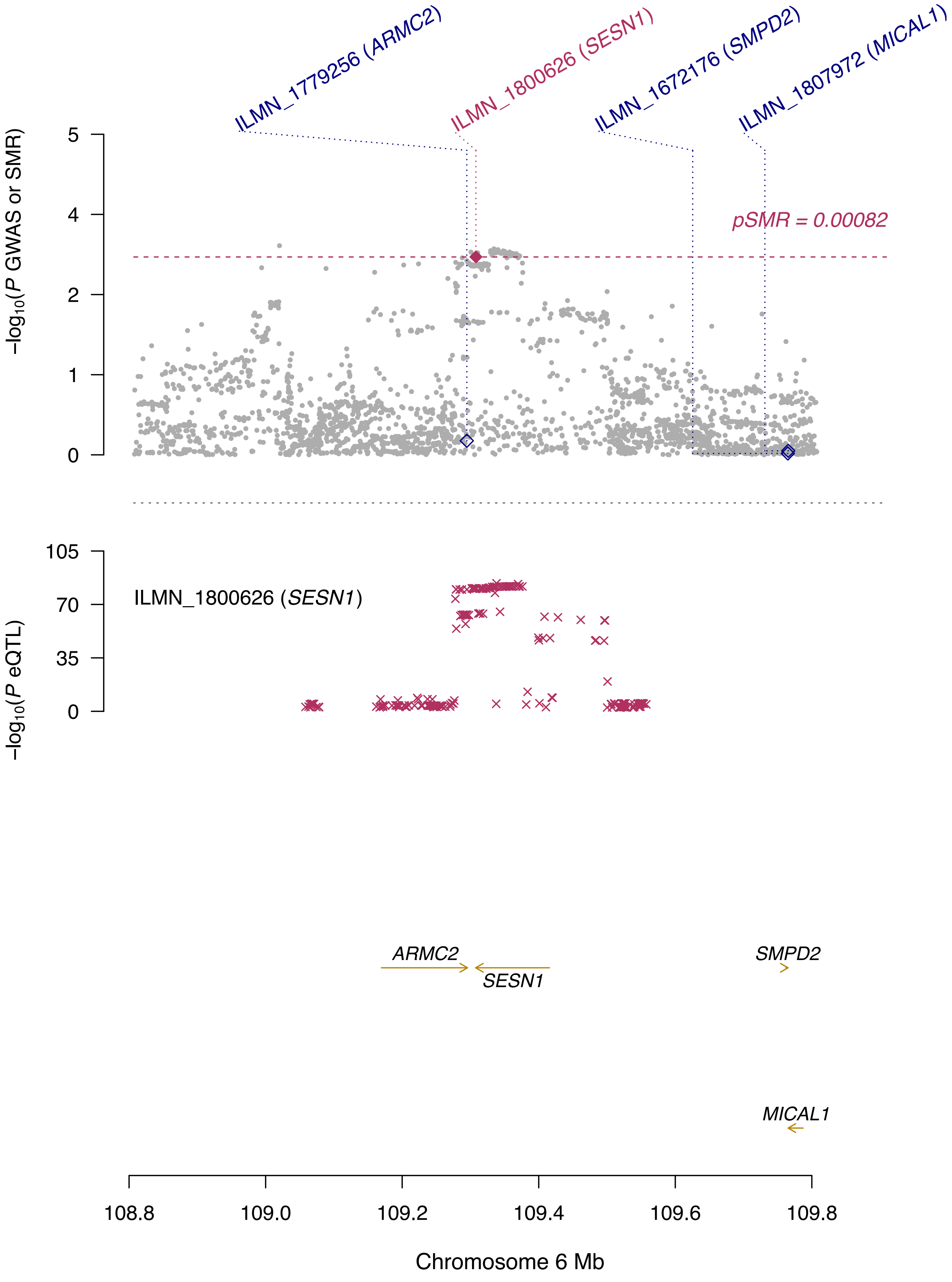
ILMN_1779256 (ARMC2)
ILMN_1800626 (SESN1)
ILMN_1672176 (SMPD2)
ILMN_1807972 (MICAL1)
pSMR = 0.00082
−log10(P GWAS or SMR)
5
4
2
1
0
105
70
35
0
ILMN_1800626 (SESN1)
−log10(P eQTL)
ARMC2
SESN1
SMPD2
MICAL1
108.8
109.0
109.2
109.4
109.6
109.8
Chromosome 6 Mb

Westra

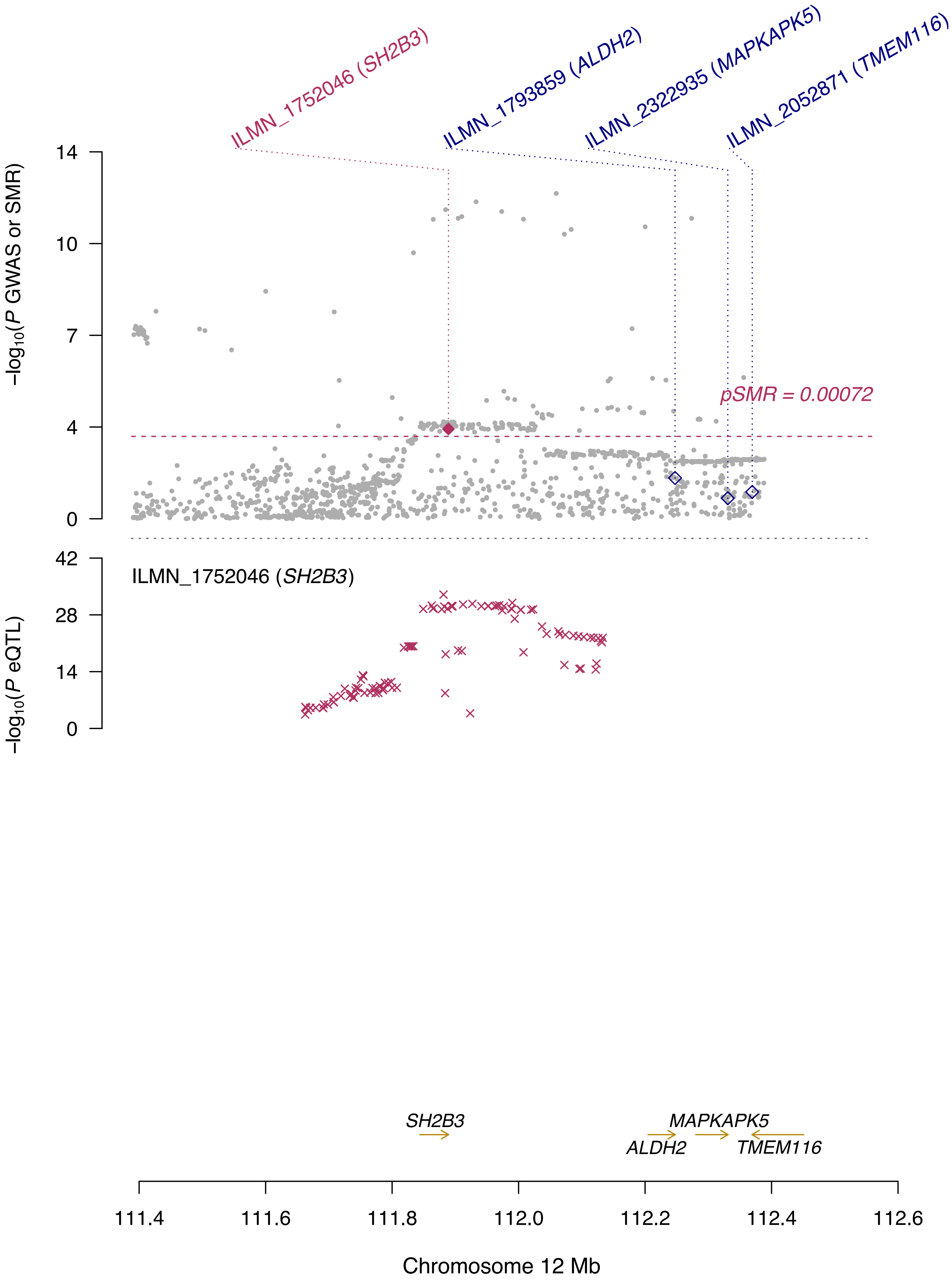

ILMN_1752046 (SH2B3)
ILMN_1793859 (ALDH2)
ILMN_2322935 (MAPKAPK5)
ILMN_2052871 (TMEM116)
14
10
7
4
0
−log10(P GWAS or SMR)
pSMR = 0.00072
42
28
14
0
−log10(P eQTL)
ILMN_1752046 (SH2B3)
SH2B3
MAPKAPK5
ALDH2
TMEM116
111.4
111.6
111.8
112.0
112.2
112.4
112.6
Chromosome 12 Mb

Westra

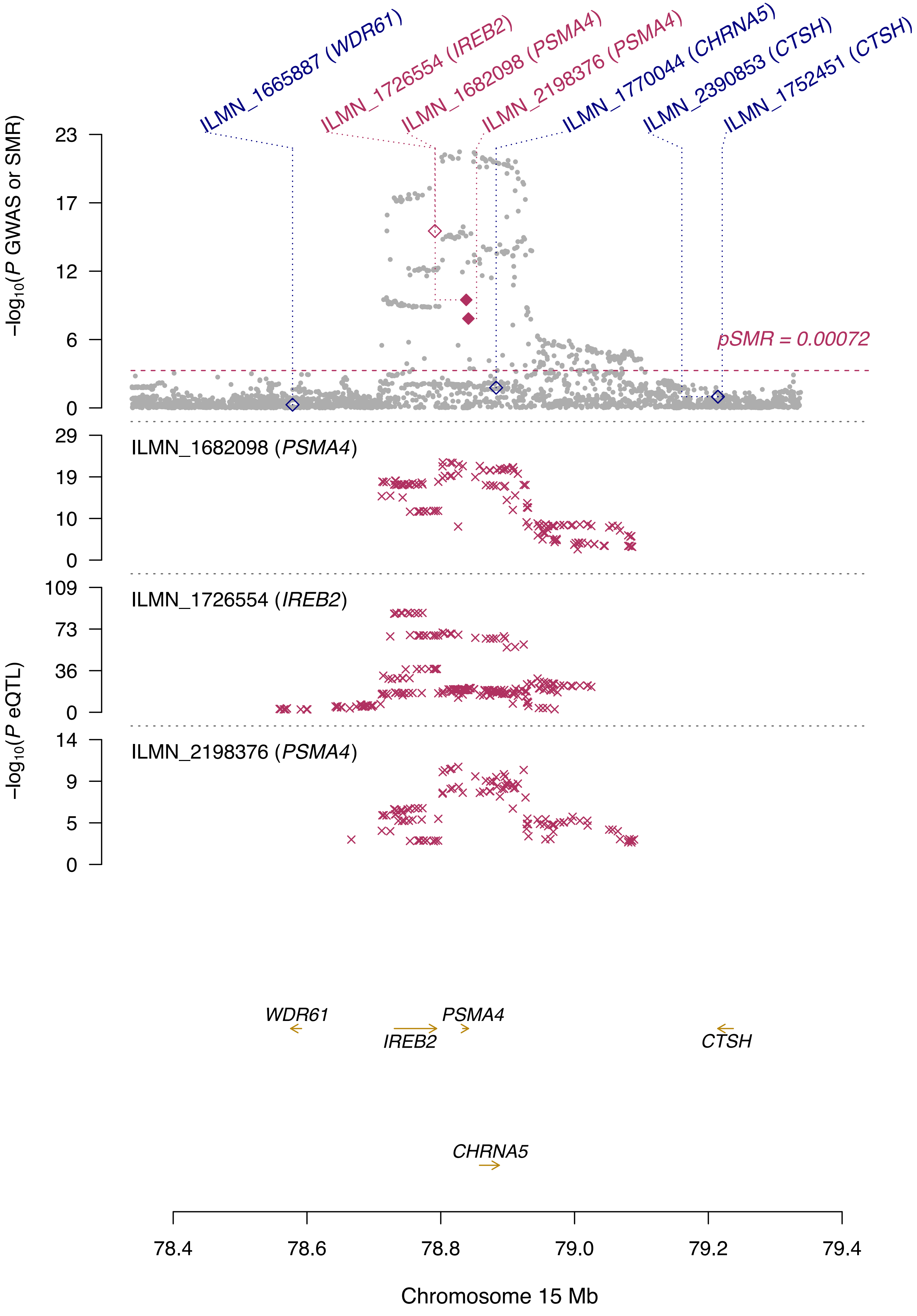
ILMN_1665887 (WDR61)
ILMN_1726554 (IREB2)
ILMN_1682098 (PSMA4)
ILMN_2198376 (PSMA4)
ILMN_1770044 (CHRNA5)
ILMN_2390853 (CTSH)
ILMN_1752451 (CTSH)
−log10(P GWAS or SMR)
23
17
12
6
0
pSMR = 0.00072
29
19
10
0
ILMN_1682098 (PSMA4)
109
73
36
0
ILMN_1726554 (IREB2)
14
9
5
0
ILMN_2198376 (PSMA4)
−log10(P eQTL)
WDR61
PSMA4
IREB2
CTSH
CHRNA5
78.4
78.6
78.8
79.0
79.2
79.4
Chromosome 15 Mb

Westra

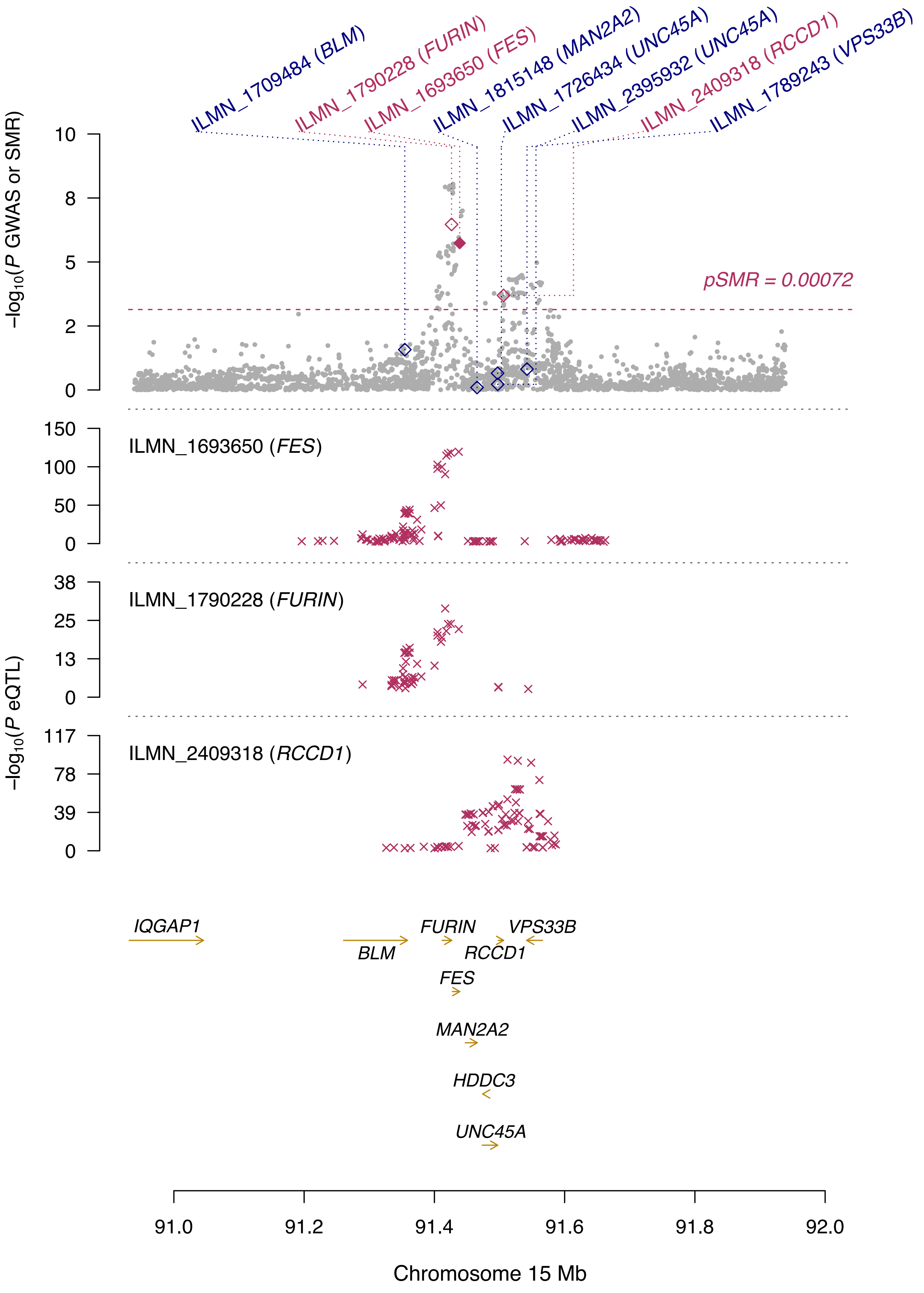

ILMN_1709484 (BLM)
ILMN_1790228 (FURIN)
ILMN_1693650 (FES)
ILMN_1815148 (MAN2A2)
ILMN_1726434 (UNC45A)
ILMN_2395932 (UNC45A)
ILMN_2409318 (RCCD1)
ILMN_1789243 (VPS33B)
−log10(P GWAS or SMR)
pSMR = 0.00072
ILMN_1693650 (FES)
ILMN_1790228 (FURIN)
ILMN_2409318 (RCCD1)
−log10(P eQTL)
IQGAP1
BLM
FURIN
RCCD1
VPS33B
FES
MAN2A2
HDDC3
UNC45A
91.0
91.2
91.4
91.6
91.8
92.0
Chromosome 15 Mb

Westra

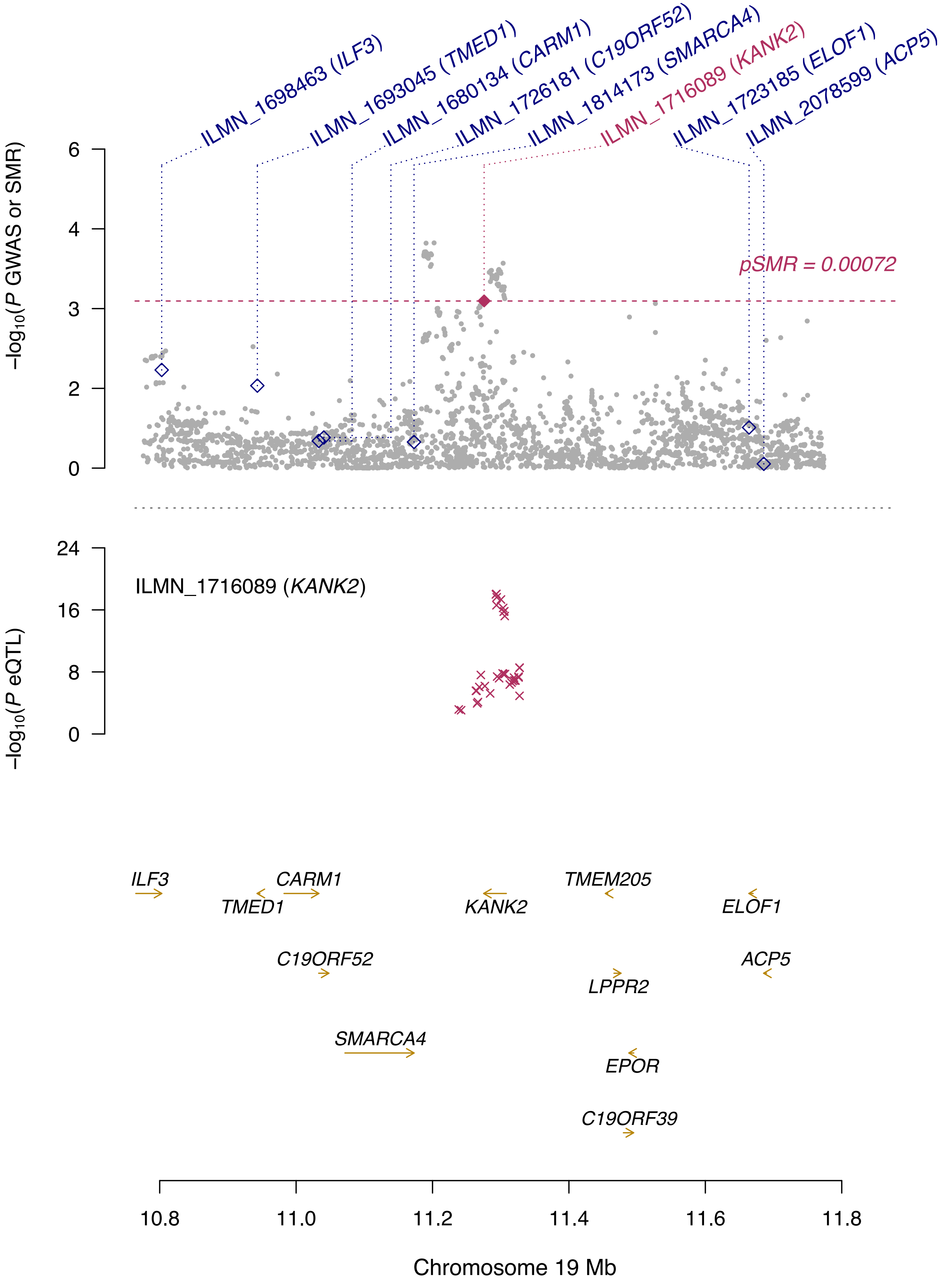

ILMN_1698463 (ILF3)
ILMN_1693045 (TMED1)
ILMN_1680134 (CARM1)
ILMN_1726181 (C19ORF52)
ILMN_1814173 (SMARCA4)
ILMN_1716089 (KANK2)
ILMN_1723185 (ELOF1)
ILMN_2078599 (ACP5)
pSMR = 0.00072
−log10(P GWAS or SMR)
0
2
3
4
6
ILMN_1716089 (KANK2)
−log10(P eQTL)
0
8
16
24
ILF3
CARM1
TMED1
KANK2
TMEM205
ELOF1
C19ORF52
ACP5
LPPR2
SMARCA4
EPOR
C19ORF39
10.8
11.0
11.2
11.4
11.6
11.8
Chromosome 19 Mb

CAGE

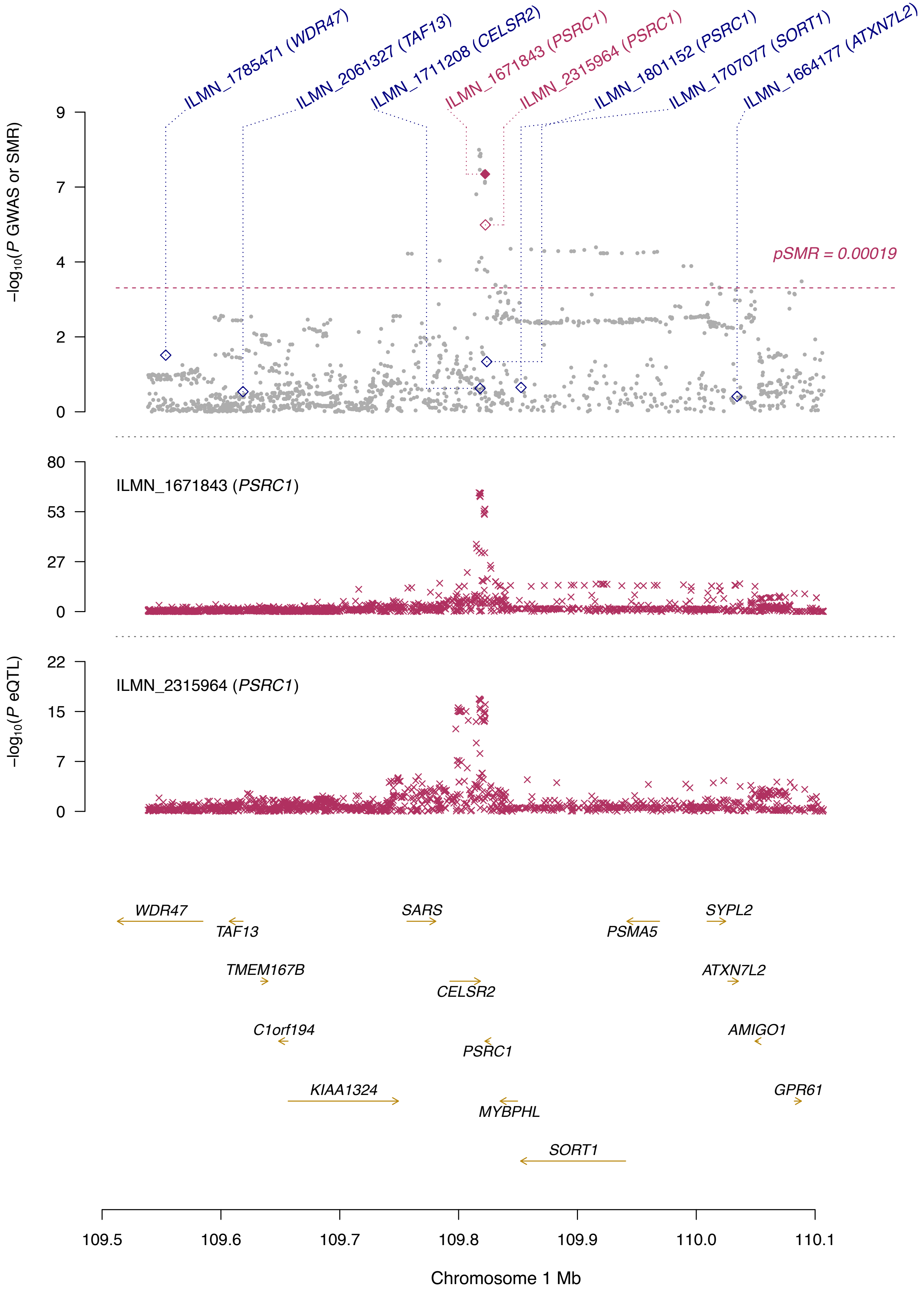
ILMN_1785471 (WDR47)
ILMN_2061327 (TAF13)
ILMN_1711208 (CELSR2)
ILMN_1671843 (PSRC1)
ILMN_2315964 (PSRC1)
ILMN_1801152 (PSRC1)
ILMN_1707077 (SORT1)
ILMN_1664177 (ATXN7L2)
−log10(P GWAS or SMR)
pSMR = 0.00019
ILMN_1671843 (PSRC1)
ILMN_2315964 (PSRC1)
−log10(P eQTL)
WDR47
TAF13
TMEM167B
C1orf194
KIAA1324
SARS
CELSR2
PSRC1
MYBPHL
SORT1
PSMA5
SYPL2
ATXN7L2
AMIGO1
GPR61
109.5
109.6
109.7
109.8
109.9
110.0
110.1
Chromosome 1 Mb

CAGE

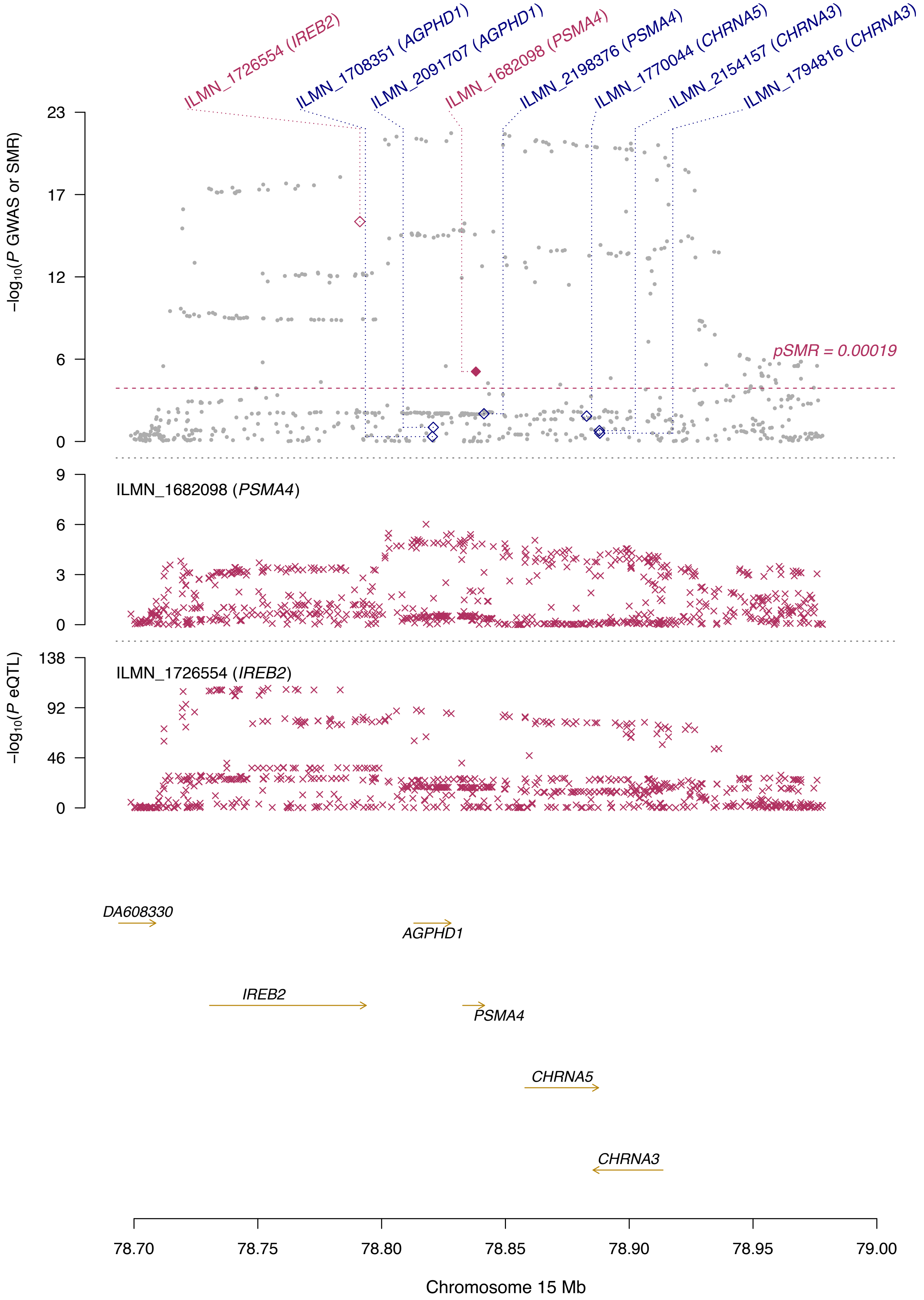
ILMN_1726554 (IREB2)
ILMN_1708351 (AGPHD1)
ILMN_2091707 (AGPHD1)
ILMN_1682098 (PSMA4)
ILMN_2198376 (PSMA4)
ILMN_1770044 (CHRNA5)
ILMN_2154157 (CHRNA3)
ILMN_1794816 (CHRNA3)
−log10(P GWAS or SMR)
23
17
12
6
0
pSMR = 0.00019
9
6
3
0
ILMN_1682098 (PSMA4)
−log10(P eQTL)
138
92
46
0
ILMN_1726554 (IREB2)
DA608330
AGPHD1
IREB2
PSMA4
CHRNA5
CHRNA3
78.70
78.75
78.80
78.85
78.90
78.95
79.00
Chromosome 15 Mb

CAGE

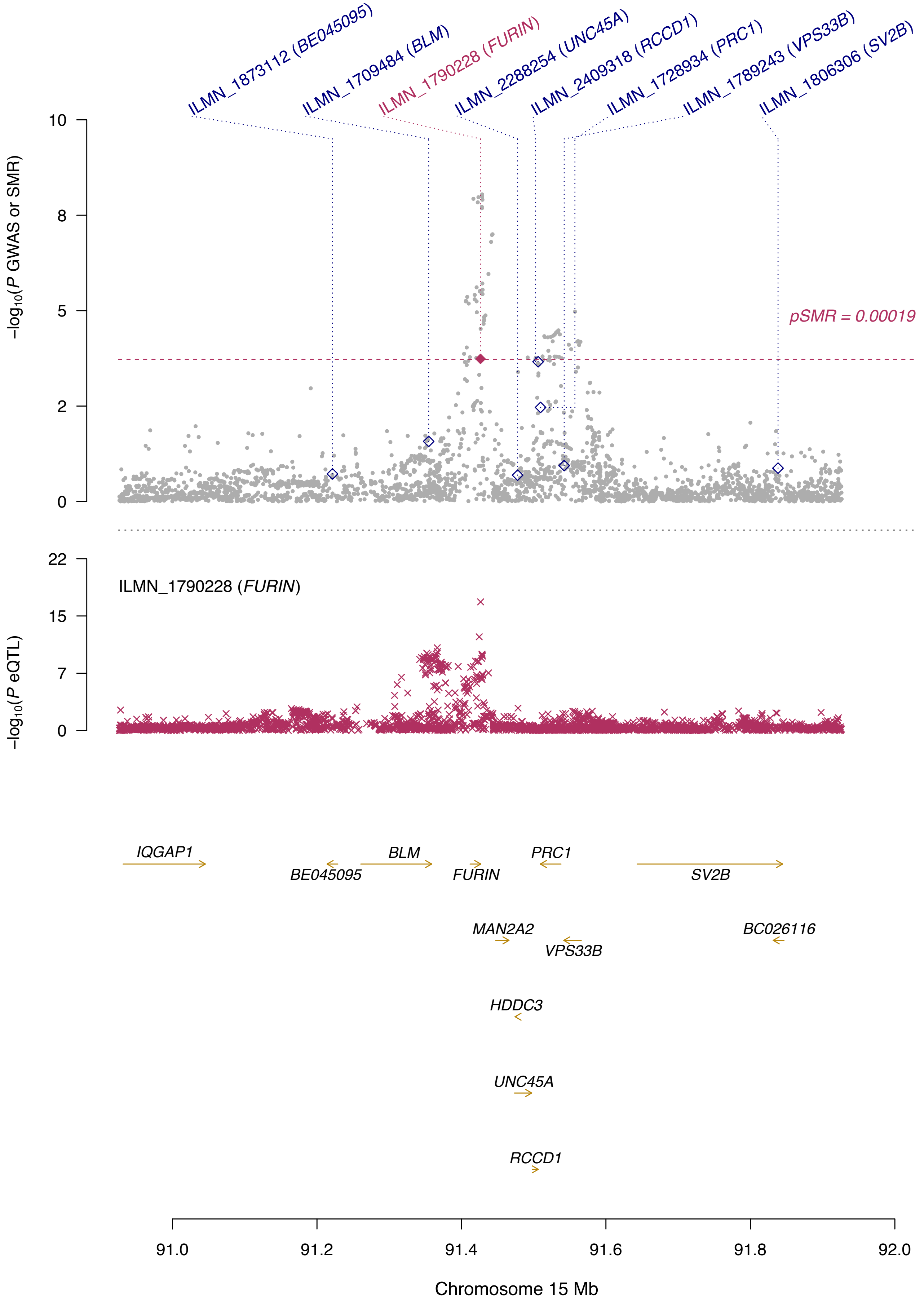
ILMN_1873112 (BE045095)
ILMN_1709484 (BLM)
ILMN_1790228 (FURIN)
ILMN_2288254 (UNC45A)
ILMN_2409318 (RCCD1)
ILMN_1728934 (PRC1)
ILMN_1789243 (VPS33B)
ILMN_1806306 (SV2B)
−log10(P GWAS or SMR)
10
8
5
2
0
pSMR = 0.00019
−log10(P eQTL)
22
15
7
0
ILMN_1790228 (FURIN)
IQGAP1
BLM
BE045095
FURIN
PRC1
SV2B
MAN2A2
VPS33B
BC026116
HDDC3
UNC45A
RCCD1
91.0
91.2
91.4
91.6
91.8
92.0
Chromosome 15 Mb
