# Supplementary material for: Genomics of 1 million parent lifespans implicates novel pathways and common diseases and distinguishes survival chances
Source: eLife. 2019 Jan 15;8:e39856. doi: 10.7554/eLife.39856 (PMC6333444; doi:10.7554/eLife.39856)
Supplement: Supplementary file 6. — This PDF contains a table and figure on the age-related gene expression analysis. [file elife-39856-supp6.pdf]

Supplementary file 6: eQTL SNPs associated with lifespan are for genes whose expression varies with age

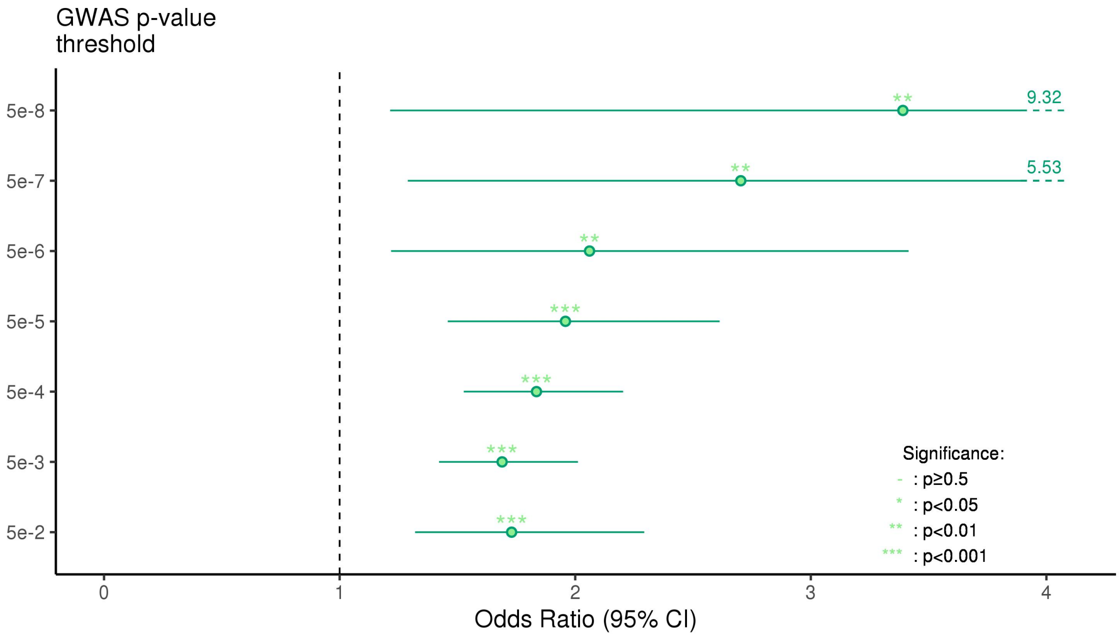

	Number of eQTLs			
Threshold	All genes	Age-related genes	OR	P
5E-8	19	9	3.39	0.0094
5E-7	36	15	2.70	0.0061
5E-6	74	26	2.06	0.0056
5E-5	241	80	1.96	5.9E-06
5E-4	800	237	1.84	7.2E-11
0.005	2078	512	1.69	9.4E-10
0.05	3083	685	1.73	3.0E-05
1	3577	755	-	-

We identified SNPs in our CES GWAS that were also eQTLs i.e. associated with the expression of at least one gene with P < 10-3 in a dataset provided to us by the eQTLGen Consortium. A total of 3,577 eQTLs after distance pruning (500 kb) were present, of which 755 were associated with genes differentially expressed with age. We used Fisher's exact test to determine, amongst the set of eQTLs, if SNPs which were associated with lifespan (at varying thresholds of statistical significance) were enriched for SNPs associated with genes whose expression is age-related. Odds ratio and 95% confidence intervals from Fisher's exact test are represented for different thresholds of statistical significance. Upper bounds of confidence interval higher than 3.7 are represented by a dotted line (respectively 9.32 and 5.53 for significance thresholds of $5x10^{-8}$ and $5x10^{-7}$). We see a significant enrichment (P < 0.05) in age-related eQTLs for all thresholds pointing out that age-related eQTLs, modulating the expression of genes differentially expressed with age, are enriched for lower than expected P value in our lifespan GWAS.
