# Supplementary material for: Genomics of 1 million parent lifespans implicates novel pathways and common diseases and distinguishes survival chances
Source: eLife. 2019 Jan 15;8:e39856. doi: 10.7554/eLife.39856 (PMC6333444; doi:10.7554/eLife.39856)
Supplement: Transparent reporting form [file elife-39856-transrepform.pdf]

eLIFE
1st Floor
24 Hills Road
Cambridge CB2 1JP, UK
P 01223 855340
W elifesciences.org
T @elife

eLife's transparent reporting form

We encourage authors to provide detailed information *within their submission* to facilitate the interpretation and replication of experiments. Authors can upload supporting documentation to indicate the use of appropriate reporting guidelines for health-related research (see EQUATOR Network), life science research (see the BioSharing Information Resource), or the ARRIVE guidelines for reporting work involving animal research. Where applicable, authors should refer to any relevant reporting standards documents in this form.

If you have any questions, please consult our Journal Policies and/or contact us: editorial@elifesciences.org.

Sample-size estimation

- You should state whether an appropriate sample size was computed when the study was being designed
- You should state the statistical method of sample size computation and any required assumptions
- If no explicit power analysis was used, you should describe how you decided what sample (replicate) size (number) to use

Please outline where this information can be found within the submission (e.g., sections or figure legends), or explain why this information doesn't apply to your submission:

As our discovery was using public data, the sample size was predetermined, and no power calculation was premade.
Similarly for replication, we took forward all GW significant discoveries to the most powered (public/previously gathered) datasets available.
The sample sets are discovered in lines 766-795.
We believe that the discovery approach is in accordance with normal practice, and the replication is in line with best practice, although many researchers now set a lower bar – polygenic replication. Their approach is akin to the summary meta-analysed result in Figure 4 ~line 256 (0.37, p<1e-13). Although we also show SNP-by-SNP results here and in our own replication Fig2a ~ line 151

Replicates

- You should report how often each experiment was performed
- You should include a definition of biological versus technical replication
- The data obtained should be provided and sufficient information should be provided to indicate the number of independent biological and/or technical replicates
- If you encountered any outliers, you should describe how these were handled
- Criteria for exclusion/inclusion of data should be clearly stated
- High-throughput sequence data should be uploaded before submission, with a private link for reviewers provided (these are available from both GEO and ArrayExpress)

Please outline where this information can be found within the submission (e.g., sections or figure legends), or explain why this information doesn't apply to your submission:

Figure 2 is essentially our single replicate. We think the single aspect is obvious. We don't think the technical/biological distinction needs to be described in this context, although our approach is akin to biological. We can include wording along these lines at revision if desired.
As is common in GWAS outlier removal did take place to remove nonsensical phenotypes, however this was done prior to and blind to the exposure, so no selective bias could have been introduced. The phenotypic QC and flitering is described ~line 770

eLife Sciences Publications, Ltd is a limited liability non-profit non-stock corporation incorporated in the State of Delaware, USA, with company number 5030732, and is registered in the UK with company number FC030576 and branch number BR015634 at the address 1st Floor, 24 Hills Road, Cambridge CB2 1JP | August 2014

Statistical reporting

- Statistical analysis methods should be described and justified
- Raw data should be presented in figures whenever informative to do so (typically when N per group is less than 10)
- For each experiment, you should identify the statistical tests used, exact values of N, definitions of center, methods of multiple test correction, and dispersion and precision measures (e.g., mean, median, SD, SEM, confidence intervals; and, for the major substantive results, a measure of effect size (e.g., Pearson's r, Cohen's d)
- Report exact p-values wherever possible alongside the summary statistics and 95% confidence intervals. These should be reported for all key questions and not only when the p-value is less than 0.05.

Please outline where this information can be found within the submission (e.g., sections or figure legends), or explain why this information doesn't apply to your submission:

We feel we have done this throughout. For our principal experiment, the phenotype transformation is described at line 184.
Sample summary descriptive statistics are shown in Table S1.~line 1709.
Tables, such as Table 1 and ST1 report effect, SE, P and the test used.

(For large datasets, or papers with a very large number of statistical tests, you may upload a single table file with tests, Ns, etc., with reference to sections in the manuscript.)

Group allocation

- Indicate how samples were allocated into experimental groups (in the case of clinical studies, please specify allocation to treatment method); if randomization was used, please also state if restricted randomization was applied
- Indicate if masking was used during group allocation, data collection and/or data analysis

Please outline where this information can be found within the submission (e.g., sections or figure legends), or explain why this information doesn't apply to your submission:

NA

Additional data files ("source data")

- We encourage you to upload relevant additional data files, such as numerical data that are represented as a graph in a figure, or as a summary table
- Where provided, these should be in the most useful format, and they can be uploaded as "Source data" files linked to a main figure or table
- Include model definition files including the full list of parameters used
- Include code used for data analysis (e.g., R, MatLab)
- Avoid stating that data files are "available upon request"

Please indicate the figures or tables for which source data files have been provided:

Each figure is supported by a supplementary data file (often the excel file).
The exception is the bulk GWAS summary statistics, which will be deposited at UK Biobank on publication, and made available on request. Again this is standard practice for GWAS
